# Revisiting the Effect of Maternal Smoking during Pregnancy on Offspring Birthweight: A Quasi-Experimental Sibling Analysis in Sweden

**DOI:** 10.1371/journal.pone.0061734

**Published:** 2013-04-17

**Authors:** Sol Pía Juárez, Juan Merlo

**Affiliations:** 1 Centre for Economic Demography, Lund University, Lund, Sweden; 2 Unit for Social Epidemiology, Department of Clinical Sciences, Faculty of Medicine, Lund University, Lund, Sweden; UCL Institute of Child Health, University College London, United Kingdom

## Abstract

Maternal smoking during pregnancy (SDP) seems associated with reduced birthweight in the offspring. This observation, however, is based on conventional epidemiological analyses, and it might be confounded by unobserved maternal characteristics related to both smoking habits and offspring birth weight. Therefore, we apply a quasi-experimental sibling analysis to revisit previous findings. Using the Swedish Medical Birth Register, we identified 677,922 singletons born between 2002 and 2010 from native Swedish mothers. From this population, we isolated 62,941 siblings from 28,768 mothers with discrepant habits of SDP. We applied conventional and mother-specific multilevel linear regression models to investigate the association between maternal SDP and offspring birthweight. Depending on the mother was light or heavy smoker and the timing of exposition during pregnancy (i.e., first or third trimester), the effect of smoking on birthweight reduction was between 6 and 78 g less marked in the sibling analysis than in the conventional analysis. Sibling analysis showed that continuous smoking reduces birthweight by 162 grams for mothers who were light smokers (1 to 9 cigarettes per day) and 226 g on average for those who were heavy smokers throughout the pregnancy in comparison to non-smoker mothers. Quitting smoking during pregnancy partly counteracted the smoking-related birthweight reduction by 1 to 29 g, and a subsequent smoking relapse during pregnancy reduced birthweight by 77 to 83 g. The sibling analysis provides strong evidence that maternal SDP reduces offspring birthweight, though this reduction was not as great as that observed in the conventional analysis. Our findings support public health interventions aimed to prevent SDP and to persuade those who already smoke to quit and not relapse throughout the pregnancy. Besides, further analyses are needed in order to explain the mechanisms through which smoking reduces birthweight and to identify other maternal characteristics that are common causes of both birthweight reduction and maternal smoking.

## Introduction

The relevance of birthweight as an indicator of a newborn's future health is well established in the literature. Birthweight has been linked to neonatal and infant mortality [1], [2] and, later in life, to intellectual impairment [3]–[5], and to specific morbidities including obesity, coronary heart diseases, type-2 diabetes, hypertension, metabolic syndrome, among others [6]–[10]. Low birthweight increases the risk of premature adult mortality [11].

In addition to some normal variation, offspring birthweight is a result of the mother's nutritional status and lifestyle [12], [13], which are influenced by the mother's cultural and socioeconomic circumstances [14]. Smoking during pregnancy (SDP) is considered the single most important determinant of decreased birthweight [15], [16]. Empirical evidence shows that SDP reduces birthweight by 200 to 377 g [17], depending on daily consumption (larger reduction for heavy smokers) [18] and the trimester in which exposure occurs (larger reduction during the last trimester) [19].

To the extent that birthweight is a mother's reproductive outcome as well as a newborn's indicator of health, genes and multiple environmental risk factors (such as socioeconomic status, ethnicity, early age of pregnancy, among others) might be correlated to both SDP and birthweight [20], and therefore, it is essential to properly establish the causal inference between these two. Randomized studies have confirmed the existence of a causal association between SDP and birthweight [21]. However, these studies are not always feasible because of ethical and practical limitations [22] and many questions remain unanswered. Therefore, most of the empirical evidence existing about the effect of SDP on child outcomes are based on observational epidemiological studies that apply conventional methods of analysis (e.g., multiple linear regressions) to adjust for potential confounding factors [23]. Because conventional studies only account for variables that are susceptible to measurement, the observed association may be confounded by unmeasured (or imperfectly measured) genetic and environmental characteristics of the mother influencing both smoking behavior and birthweight outcome [24]. Moreover, the standard design normally does not consider the existence of paired cases in the sample (e.g., siblings), which violate the assumption of independence of observations. In this context, family designs have been applied to population-based registries providing robust quasi-experimental analyses [25]–[30]. The idea behind family designs is to approximate a counterfactual situation by examining the effects of different exposures (for e.g. smoking vs non-smoking) on individuals who are genetically very similar (e.g. siblings) and share similar physical and social environments [31], [32]. The possibility of cancelling out unknown but common sibling characteristics provides the opportunity to strength causal inference in comparison to conventional analyses. [23], [33]. Moreover, these analyses properly deal with paired observations by providing intra-maternal estimations.

A previous study applying twin designs tried to elucidate causal relations between parents' characteristics and child outcomes. This analysis observed the existence of a potentially causal association between SDP on birthweight [24]. However, while this study is of fundamental relevance, the observed effect of SDP on birthweight was only an ancillary finding used to illustrate the potential of the family design for causal inference rather than to provide a new insight into the effect of SDP on birthweight. Besides, Donofrio's study covers a very large temporal window (1915–1980), which might affect the validity of the estimations as the author himself recognizes [24].

In Sweden, to the best of our knowledge, there is one study exploring the effect of SDP on birthweight using sibling data [34]. However, this investigation does not qualify as a quasi-experimental intra-familiar sibling analysis because it compares women who have smoked in two successive pregnancies to women who have never smoked in any pregnancy. The aforementioned study is just a conventional analysis which, by comparing different women, overlooks the fact that SDP is not evenly distributed across the population and, therefore, that mothers who smoke during pregnancy probably differ from those who do not smoke.

Against this background, we aim to revisit the effect of SDP on birthweight. We perform the analyses on the Swedish Birth Register and apply a quasi-experimental sibling design that allows us to account for unknown genetic and environmental confounders and thus to provide stronger causal evidence than previous conventional analyses [20], [21]. Using this approach, we investigate the effect of smoking in the 1^st^ (10th to 12th gestational weeks) and 3^rd^ trimesters (30th to 32nd gestational weeks) of pregnancy. Additionally, we disentangle the effects of continuous, quitting and relapsing smoking during pregnancy, distinguishing between light and heavy smoking habits.

## Materials and Methods

### Study population

We based our study on the Swedish Medical Birth Register (MBR), which records approximately 99% of all births in the country [35]. From the MBR, we obtained information on all babies born in Sweden between 2002 and 2010 (*n* = 938,932). Then, we selected the 677,922 singletons who were born alive and at full term to native Swedish mothers. We restricted the sample to babies born at term (≥37 gestational weeks) to avoid possible bias due to the fact that smoking has been identified as a cause of preterm birth [36] and because a considerable proportion of mothers who have pre-term babies do not provide information on smoking in the 3^rd^ trimester.

**Figure 1:**
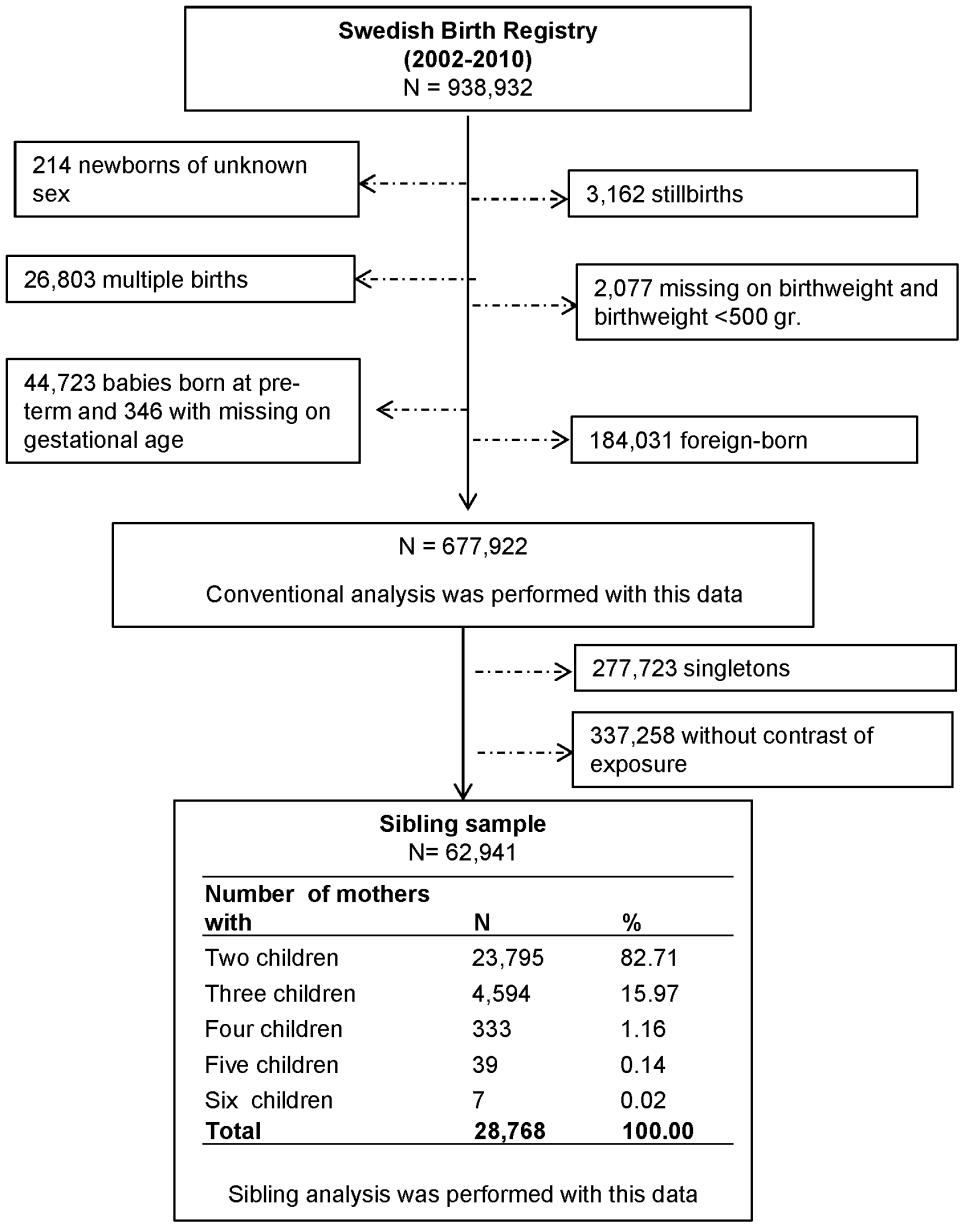
Flow diagram showing the individuals excluded from the study population and the individuals included in the study samples analyzed to investigate the effect of maternal smoking during pregnancy on offspring birthweight.

For the subsequent sibling analysis based on stable family contexts (that is to say, a design which assumes a stable environment but a discordant exposure of each sibling) [37], we performed a further selection that included only mothers with discordant smoking habits between pregnancies. In other words, we included women who had at least one pregnancy during which they smoked and at least one other pregnancy during which they did not smoke. This procedure produced 62,941 discordant siblings of 28,768 mothers (Figure 1).

### Assessment of variables

The study outcome variable was birthweight in grams (g). Information on smoking habits during pregnancy was self-reported and assessed at the first antenatal visit (i.e., between gestational weeks 10 and 12) and at the 3^rd^ trimester (i.e., between gestational weeks 30 and 32) by means of a questionnaire administered by the midwife. Mothers were classified as light (1 to 9 cig/day) or heavy smokers (>9 cig/day) according to the reported number of cigarettes smoked per day. We re-categorized the smoking information to include all possible exclusive categories between light and heavy smokers, non-smoking, and missing values in the 1^st^ and 3^rd^ trimester (see Table 1 for more detailed information). In the analyses, we used non-SDP as the reference category.

**Table 1:** Characteristics of the children and the children's mothers in the two samples.

	Whole sample	%	Sibling sample	%	Excluded sample	%
**Mother's characteristics**						
**Mothers' age (yrs)**						
<20	18,823	2.78	2,037	3.24	9,819	6.49
20–24	68,642	10.13	7,605	12.08	23,927	15.82
25–34*	450,783	66.49	42,045	66.80	94,056	62.18
35–40	138,898	20.49	11,219	17.82	23,270	15.38
>40	745	0.11	32	0.05	174	0.12
Missing values	31	0.00	3	0.00	22	0.01
**Mothers' marital status**						
Cohabiting with father*	615,095	90.73	46,705	74.2	131,006	86.61
Single	9,073	1.34	669	1.06	4,185	2.77
Other family situation	22,086	3.26	1,775	2.82	9,991	6.60
Missing values	31,668	4.67	13,792	21.91	6,086	4.02
**Complications during pregnancy**						
**Diabetes** (yes*/no)	3,431	0.51	240	0.38	797	0.53
**Urinary problems** (yes*/no)	99,086	14.62	7,852	12.48	24,189	15.99
**Hypertension** (yes*/no)	2,976	0.44	200	0.32	661	0.44
**Snus during pregnancy**						
Never used snus*	620,397	91.51	40,532	64.40	138,835	91.78
Occasionally used snus	10,927	1.61	1,198	1.90	2,640	1.75
Used snus missing/missing used snus	17,359	2.56	5,548	8.81	4,482	2.96
Missing values	29,239	4.31	15,663	24.89	5,311	3.51
**Newborn's characteristics**						
**Birth order**						
1*	306,890	45.27	24,047	38.21	151,268	100.00
2	255,215	37.65	27,298	43.37		
3	86,587	12.77	8,589	13.65		
>4	29,230	4.31	3,007	4.78		
**Newborn's gender**						
Female*	330,105	48.69	30,319	48.17	73,904	48.86
Male	347,817	51.31	32,622	51.83	77,364	51.14
**Gestational age (weeks)**						
Term*	626,631	92.43	58,604	93.11	136,966	90.55
Posterm	51,291	7.57	4,337	6.89	14,302	9.45
**Birth weight mean (N; standard error)**	3631 (677,922; 489)		3651 (6,241; 485)		3531 (151,268; 482)	

We adjusted all models for gestational age, use of Swedish snus during pregnancy, birth order, sex, mother's age, complications during pregnancy, and marital status (see Table 1 for detailed information on the categorization of the variables and the categories used as references in the comparisons).

The Swedish National Board of Health and Welfare constructed the database using individual-level information and provided the database to us after encrypting the individual identification numbers. The database was approved by the regional ethical review board in southern Sweden. The board did not require explicit informed consent from the women.

### Statistical analyses

In a first step, we applied a conventional multiple linear regression to estimate the association between maternal SDP and offspring birthweight on the 677,922 singletons born alive to Swedish mothers (Figure 1). The variables included in the model are indicated in Table 1. We calculated the beta coefficient and confidence intervals (CIs) at 95%.

In a second step, we performed a multilevel linear regression [38], [39] with siblings at the first level and mothers at the second level. The purpose of this analysis was to obtain mother-specific regression coefficients [40]. By including a random term for the mother, the multilevel regression analysis adjusted for unknown genetic and environmental factors related to every mother. Additionally, in an attempt to reduce temporal confounding factors (e.g., maternal variables changing between pregnancies) [33], we adjusted the model with the same independent variables described in the previous section (table 1). We also calculated the Variance Partitioning Coefficient (VPC), which is a measure of clustering that allows us to estimate how much of the total individual variance in birthweight is at the maternal level.

In the multilevel regression, we used Markov Chain Monte Carlo (MCMC) [41] method with orthogonal parameterization to estimate the parameters and the statistical package MLWIN 2.23 (Centre for Multilevel Modelling, University of Bristol, UK).

## Results

The socio-demographic and clinical characteristics of singletons born alive to Swedish mothers and of the subpopulation of mothers with at least two siblings and discordant smoking habits between pregnancies are presented in Table 1. Overall, the characteristics of the two populations studied were very similar. However, compared with the complete dataset, the sibling sample shows a higher proportion of mothers with missing information regarding marital status and the use of Swedish snus during pregnancy. Furthermore, as expected, the complete dataset has a larger proportion of first-order newborns because this dataset includes children without siblings born in the study period, and these children are excluded from the sibling sample. In comparison to the excluded sample (which only contains single babies without siblings), the sibling sample shows a lower proportion of young mothers (20 years or younger) and post-term babies as well as a larger proportion of mothers with missing data on marital status and on snus use.

Table 2 shows the results of the conventional and sibling analyses regarding the effect of SDP on birthweight. The results are presented to assess four domains: (i) the effect of continuous smoking during pregnancy, (ii) the effect of quitting smoking or (iii) of subsequently relapsing during the pregnancy, and (iv) the influence of missing information on smoking habits in the 1^st^ and/or the 3^rd^ trimester of pregnancy.

**Table 2:** Association between categories of smoking during pregnancy (SPD) and offspring's birthweight by different designs.

Categories of smoking	Conventional analysis				Sibling design	
	N (%)	β	CI-95%	N (%)	β	CI-95%
**Non-SDP**						
(Intercept/reference)	578,583 (85.35)	3585	[3583 3587]	32,980 (52.4)	3607	[3600 3614]
**Continues smoking**						
Light-light	19,910 (2.94)	−221	[−227 −215]	1,988 (3.16)	−162	[−178 −147]
Heavy-heavy	5,613 (0.83)	−303	[−313 −292]	189 (0.40)	−226	[−274 −179]
Light-heavy	2,842 (0.42)	−265	[−279 −250]	171 (0.27)	−259	[−309 −209]
Heavy-light	4,007 (0.59)	−254	[−266 −242]	227 (0.36)	−194	[−238 −151]
**Quitting smoking**						
Light-non smoking	10,445 (1.54)	−47	[−55 −40]	2,757 (4.38)	−29	[−42 −16]
Heavy-non smoking	1,380 (0.20)	−79	[−100 −58]	226 (0.36)	−1	[−46 44]
**Relapsing smoking**						
Non smoking-light	4,023 [0.59]	−129	[−142 −117]	1,073 (1.70)	−77	[−97 −57]
Non smoking-heavy	480 (0.07)	−142	[−177 −108]	128 (0.20)	−83	[−140 −25]
**Missing information**						
Non smoking- missing	10,803 (1.59)	−2	[−11 7]	4,681 (7.44)	−7	[−31 −7]
Missing-missing	29,200 (4.31)	0.1	[−64 63]	15,668 (24.89)	7	[−7 22]
Missing-light	265 (0.04)	−210	[−259 −162]	31 (0.01)	−40	[−158 77]
Missing-heavy	95 (0.01)	−333	[−414 −257]	3 (0.00)	−505	[−891 −131]
Missing- non smoking	3,602 (0.53)	−14	[−30 2]	1,930 (3.07)	−16	[−138 108]
Light-missing	4,924 (0.73)	−191	[−203 −179]	792 (1.26)	−87	[−113 −61]
Heavy-missing	1,750 (0.26)	−282	[−301 −263]	97 (0.15)	−141	[−206 −75]

All models were adjusted for gestational age, marital status, maternal age, birth order, sex of the newborn, complications during pregnancy (i.e., diabetes, hypertension and urinary problems), and Swedish snus use.

### (i) Continuous smoking during pregnancy

The conventional analysis showed that heavy smokers experienced the highest reduction in birthweight (303 g), followed by those who were light smokers in the 1^st^ trimester and those who became heavy smokers in the 3^rd^ trimester (265 g reduction). This gradient was completed by heavy smokers in the 1^st^ trimester who became light smokers in the 3^rd^ trimester (254 g reduction) and those who remained light smokers throughout the entire pregnancy (221 g reduction).

The results from the sibling analysis were analogous to those from the conventional analysis, although the birthweight reduction in the sibling analysis was smaller for almost all categories of smoking than in the conventional analysis. The greatest differences between designs were observed in the categories of heavy-heavy and light-light, with reductions of 226 and 162 g, respectively. After applying the sibling analysis, the effect of heavy-light smokers was 60 g smaller than in the conventional analysis. In contrast, the effect of light-heavy smokers was greater than in the conventional analysis (259 g reduction).

### (ii) Quitting smoking during pregnancy

The conventional analysis indicated that stopping smoking after the 1^st^ trimester reduces birthweight, although this result depends on the number of cigarettes smoked per day. The birthweight reduction in offspring of light smoking mothers who quit smoking after the 1^st^ trimester was 47 g, whereas the reduction was 79 g for mothers who were heavy smokers. The sibling analysis showed that the reduction was smaller and less pronounced for heavy smokers (29 and 1 g, respectively).

### (iii) Relapsing smoking during pregnancy

The conventional analysis showed reduced birthweight for the offspring of mothers who did not smoke in the 1^st^ trimester but subsequently relapsed during pregnancy. These offspring had a 142 g reduction if their mothers became heavy smokers in the 3^rd^ trimester and a reduction of 129 g if their mothers became light smokers. We found analogous effects in the sibling analyses, but in absolute values, the reduction was substantially less apparent than in the conventional analysis (i.e., 83 g for the category of non smoking-heavy smokers and 77 of non smoking-light smokers).

### (iv) Missing information on smoking habits

Birthweight reduction was considerable when mothers were smokers (either light or heavy) in one of the trimesters and had missing information in the other trimester. These results vary in the sibling analysis, but these differences could be at least partly explained by the relatively low number of children in some categories. The conventional analysis shows that mothers who did not report information on smoking in any trimester of the pregnancy (i.e., missing-missing) and those who reported not having smoked in one trimester and had missing information in the other trimester (i.e., non smoking-missing and missing-non smoking) did not differ from those who never smoked. This result is confirmed in the sibling analysis.

## Discussion

Using a conventional linear regression analysis, our study was able to replicate previous findings [17], [42], indicating that maternal smoking during pregnancy reduced offspring birthweight. We also observed that the specific effect of smoking during pregnancy depends on the number of cigarettes smoked per day (with a greater effect among heavy smokers) and the timing of the exposure (with a greater effect at the end of gestation). These results were confirmed with a quasi-experimental sibling analysis, which provides stronger causal evidence than the conventional analysis. However, we also observed that the offspring birthweight reduction was smaller in the sibling analysis than in the conventional analysis, which suggests that conventional analyses were somewhat confounded by unknown maternal characteristics related to both SDP and offspring birthweight.

From a public health perspective, the sibling analysis provides strong evidence that confirms the negative effects of smoking during pregnancy. Our results support policies aimed not only at preventing smoking during pregnancy, but also directed at persuading pregnant women to quit smoking and to avoid relapsing

To the extent that our results suggest the existence of a causal association, we need to develop further research aiming at identifying the specific mechanisms through which smoking reduces birthweight. Most studies so far agree that cigarette smoke contains toxic substances such as carbon monoxide (CO), metals and nicotine [43] which can be responsible for birthweight reduction. However, it is difficult to isolate their effects and there are still a number of chemical constituents and additives which remain untested for developmental toxicity [15]. A recent animal study showed that the reduction in fetal weight in rats is due to CO toxicity and not nicotine toxicity [44]. However, further studies in humans are needed to confirm this finding because humans may inhale smoke and metabolize toxins in a different way than in the controlled experimental environment of animal studies [28].

Our results also demonstrate that conventional analyses are to some extent confounded by inter-mother differences in unknown genetic, behavioral, and/or environmental factors. Although these unobserved variables do not fully explain the association existing between SDP and birthweight reduction, these are important enough to be considered. For example, it is possible that mothers who smoke are limited in their options to maintain a lifestyle that promotes good general health (e.g., by practicing sports and observing good dietary habits), and these conditions reduce offspring birthweight more sharply. Therefore, any preventive strategy against birthweight reduction that focuses only on persuading women to quit smoking in terms of personal responsibility might to some extent “blame the victim” without considering the circumstances that are linked to both smoking during pregnancy and birthweight reduction in the offspring. Therefore, further analysis is needed to identify these circumstances or other hazardous exposures like alcohol or environmental pollution, which, in addition to preventing smoking, may be suitable targets for public health interventions. Our study identifies the existence of a certain confounder related to maternal characteristics. However, we are yet to disentangle the gene-environmental correlation involved [33].

The application of a quasi-experimental sibling analysis is a clear strength of our study. This design provides opportunities to investigate the causal effect of smoking on birthweight by studying, *ceteris paribus*, the effects of different exposures (smoking/non-smoking) on the birthweight of individuals who are very similar (e.g., siblings). Moreover, in comparison to the excluded sample (without siblings), the sibling sample is highly representative with regard to maternal and child characteristics, which guarantees that our findings can be generalized [33].

We observed that the intra-maternal clustering (VPC) of offspring birthweight was 49%. In other words, a considerable share of the total individual variance in birthweight was at the maternal level, which supports the suitability of the sibling design for the study of causal associations. Against this background, the sibling analysis confirmed that the adverse effect of smoking was not mediated by other factors, such as the mother's education, although mothers with a high educational level were more likely to not smoke during pregnancy [18], [45].

Our study has a number of strengths in addition to the quasi-experimental approach. First, our analyses are based on a national medical registry containing standardized information and covering the entire Swedish population. Second, because giving birth at home is very unusual in Sweden, nearly all births are registered in the MBR. Finally, we had information spanning a relatively long period (2002–2010), with two measurements of smoking habits at different stages of gestation.

Our investigation also had some limitations. The validity of the information on smoking habits during pregnancy was self-reported by the mothers, which might to some extent bias the result by random measurement error of exposure [46]. Because SDP is a well-known health hazard, social control might lead to underreporting of true smoking habits and to underestimating of the effects of smoking. Therefore, it is possible that the light smoker group may also include heavy smokers, and the non-smoking group may include some smokers. The unexpected smaller reduction observed in the categories of light-heavy (in comparison to heavy-heavy) and non smoking-heavy (in comparison to and non smoking-light) might be explained by this. However, collecting our information on smoking in two different moments of the pregnancy may reduce the bias of classifying a true smoker as non-smoker. A study conducted in Sweden comparing self-reported nicotine exposure and plasma levels of cotinine in early and late pregnancy concluded that self-reported smoking information had acceptable validity [47]. Besides, we cannot identify the specific number of cigarettes smoked beyond the light and heavy classification that the MBR provides or to know the specific week in which the mother quitted or relapsed smoking. Therefore, we are not able to know whether the observed effects are mainly happening at the beginning of the third trimester or at the end of the second.

Despite the sibling design and the adjustment for temporal confounding, we cannot exclude the existence of residual confounding. For example, siblings are only matched based on the fact that they share the same mother. However, we had no information about fathers, so some of the siblings might be half siblings (this information was not available in our dataset). Moreover, it is possible that the association between SDP and birthweight is confounded by smoking in the father, influencing the child through passive smoking in the mother or smoke exposure among those who do not smoke or quit. This is important because sibling analyses are effective to control for unobserved characteristics and genetic influences which siblings have in common. However, sibling analyses cannot rule out any unmeasured variables that simultaneously vary between siblings [46].

Moreover, we adjusted for snus use, but we did not have information on other forms of exposure, such as nicotine replacement therapy (e.g., nicotine patches, gums, sprays, inhalers) or passive smoking. Previous studies have shown that nicotine replacement therapy does not have a significant association with birthweight reduction (except when different products are used simultaneously) [48]. However, many studies support the harmful effects of passive smoking on birthweight [49], [50].

Another limitation of our study was the relatively large amount of missing information on smoking habits. However, we had information for both the 1^st^ and the 3^rd^ trimesters of pregnancy, which allowed us to identify different categories of missing information, making the missing data more informative.

Birthweight reduction was considerable when mothers were (either light or heavy) smokers in one trimester, and these results were similar in the sibling analysis. These findings highlight the importance of more than one observation of smoking habits during pregnancy to better identify at-risk babies. Our results suggest that the missing values are not randomly distributed with respect to smoking habits. Therefore, further analysis is required to identify the characteristics of the mothers with missing information on smoking habits.

In summary, our study was able to replicate previous conventional linear regression analyses indicating that maternal smoking during pregnancy reduces offspring birthweight [17], [42]. We confirmed that birthweight reduction depends on whether the mother is a light or heavy smoker and on the trimester of exposure. The sibling analysis not only confirms these effects, but also provides strong evidence for a causal association between SDP and birthweight. We need, however, further research aimed at disentangling the mechanisms underlying the smoking effect. However, the sibling analysis also shows that the effect of smoking on offspring birthweight is smaller than previously reported, which implies that to some degree, conventional analyses appear confounded by between-mother differences in unknown genetic, behavioral and/or environmental factors that are a common cause of both maternal SDP and offspring birthweight. In this regard, it seems relevant to identify these circumstances because, in addition to preventing smoking, they may be suitable targets for public health interventions aimed at increasing offspring birthweight.
